# Supplementary material for: Repeated intravenous administration of hiPSC-MSCs enhance the efficacy of cell-based therapy in tissue regeneration
Source: Commun Biol. 2022 Aug 25;5:867. doi: 10.1038/s42003-022-03833-8 (PMC9411616; doi:10.1038/s42003-022-03833-8)
Supplement: Supplementary file 2 — Supplementary Information [file 42003_2022_3833_MOESM2_ESM.pdf]

Repeated Intravenous Administration of hiPSC-MSCs Enhance the Efficacy of Cell-based Therapy in Tissue regeneration

Si-Jia Sun;[1,2*] Fei Li;[2*] Ming Dong;[3] Wei-Hao Liang;[4] Wing-Hon Lai;[2] Wai-In Ho;[2] Rui Wei;[2] Yan Huang;[5] Song-Yan Liao;[2,6#] Hung-Fat Tse.[2, 6, 7, 8#]

[1]Division of Cardiology, Department of Medicine, The First Affiliated Hospital of Soochow University, Soochow University, Suzhou, China; [2]Cardiology Division, Department of Medicine, Queen Mary Hospital, the University of Hong Kong, Hong Kong SAR, China; [3]Bioland Laboratory, Guangzhou Regenerative Medicine and Health Guangdong Laboratory, Guangzhou, China; [4]Department of Cardiology, The First Affiliated Hospital of Sun Yat-Sen University, Guangzhou, China; [5]Anhui Provincial laboratory of inflammatory and immunity disease, Institute of Innovative Drugs, School of Pharmacy, Anhui Medical University, Hefei, China; [6]Shenzhen Institutes of Research and Innovation, the University of Hong Kong, Hong Kong SAR, China; [7]Hong Kong-Guangdong Joint Laboratory on Stem Cell and Regenerative Medicine, the University of Hong Kong and Guangzhou Institutes of Biomedicine and Health, Hong Kong SAR, China; [8]Department of Medicine, Shenzhen Hong Kong University Hospital, Shenzhen, China;

Short title: Mesenchymal Stromal Cell-based Therapy

* SJ Sun and Fei Li contributed equally to this work and are co-first authors.

# SY Liao and HF Tse contributed equally to the supervision of this work and are co-corresponding authors.

Address for correspondence:

Hung-Fat Tse, MD, PhD; or Song-Yan Liao, PhD;

Department of Medicine, The University of Hong Kong, Queen Mary Hospital, Hong Kong, China. Tel.: (852) 2255-4694; Fax: (852) 2818-6304

E-mail: hftse@ hku.hk; or lsy923@hku.hk

Supplemental materials

Supplementary Figure 1. Biodistribution of intravenously administrated hiPSC-MSCs.

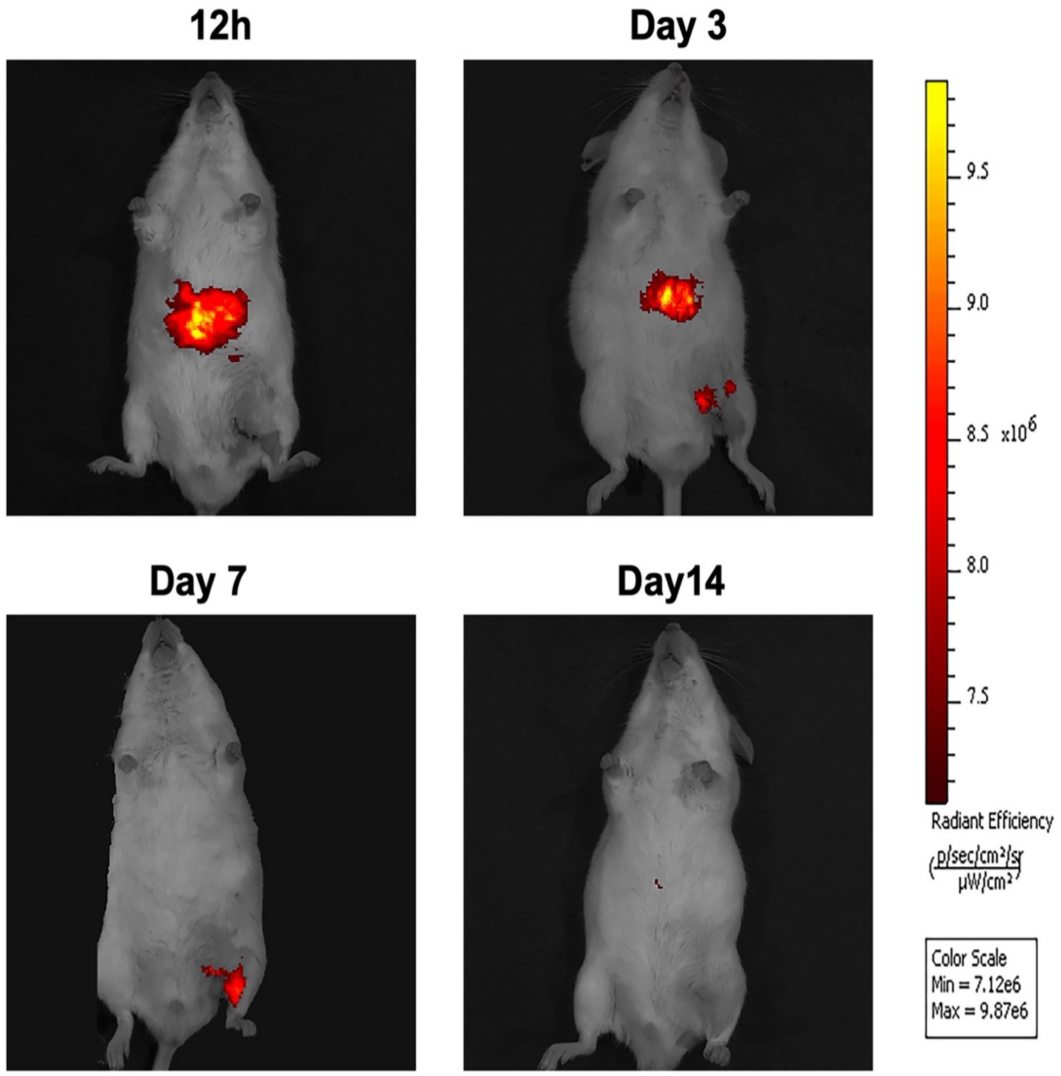

To determine cell engraftment after a single intravenous cellular infusion, 3 mice receiving intravenous DiR-labeled hiPSC-MSC infusion after induction of ischemia were scanned under epi-fluorescent imaging at 12h and on day 3, 7 and 14. Most hiPSC-MSCs engrafted into the liver 12h after intravenous cellular infusion. The engrafted hiPSC-MSCs gradually migrated into ischemic limbs at day 3 and had disappeared by day 14.

Supplementary Figure 2. Repeated intravenous administration of hiPSC-MSCs prolonged the survival of transplanted hiPSC-MSCs.

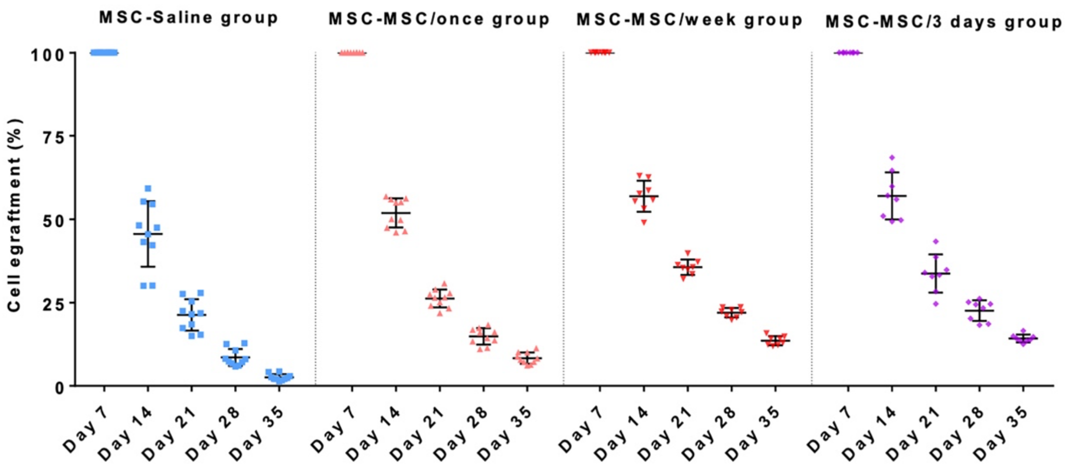

The estimated survival rates at day 14, 21, 28 or 35 was calculated as the percentage of the radiant efficiency at day 14, 21, 28 or 35 versus the percentage of the averaged radiant efficiency after intravenous transplantation.

Supplementary Figure 3. Repeated intravenous administration of hiPSC-MSCs increased anti-inflammatory cytokines and decreased inflammatory cytokines.

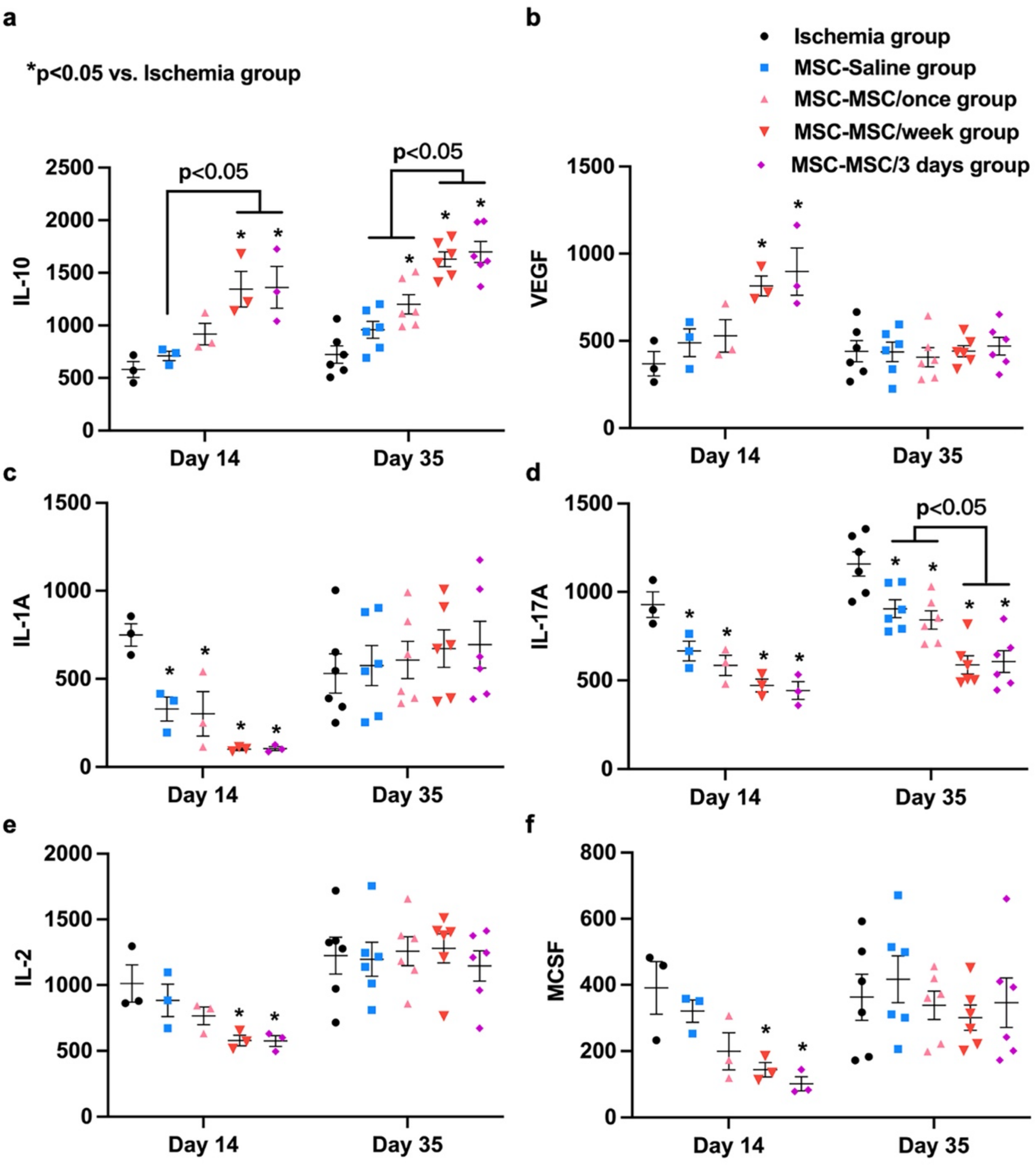

A mouse inflammatory factors array was used to evaluate the changes of cytokines. Repeated Intravenous administration of hiPSC-MSCs in the MSC-MSC/week and MSC-MSC/3 days groups improved IL-10 from day 14 onwards relative to the ischemia group. On day 35, intravenous administration of hiPSC-MSCs in the MSC-MSC/once group significant increased IL-10 compared with the ischemia group. Moreover, repeated Intravenous administration of hiPSC-MSCs in the MSC-MSC/week and MSC-MSC/3 days groups further increased IL-10 compared with the MSC-MSC/once group (**a**). Repeated Intravenous administration of hiPSC-MSCs in the MSC-MSC/week and MSC-

MSC/3 days groups improved VEGF on day 14 relative to the ischemia group (**b**). On day 14, IL-1A and IL-17A significantly decreased in the MSC-Saline, MSC-MSC/once, MSC-MSC/week and MSC-MSC/3 days groups compared with the ischemia group (**c-d**). Moreover, IL-17A further decreased in the MSC-MSC/week and MSC-MSC/3 days groups compared with the MSC-Saline and MSC-MSC/once groups (**d**). IL-2 and MCSF significantly decreased in the MSC-MSC/week and MSC-MSC/3 days groups compared with the ischemia group on day 14 and no significant difference was observed among all five group on day 35 (**e-f**).

Supplementary Figure 4. Repeated intravenous administration of hiPSC-MSCs further reduced NK cells and continuously increased Tregs.

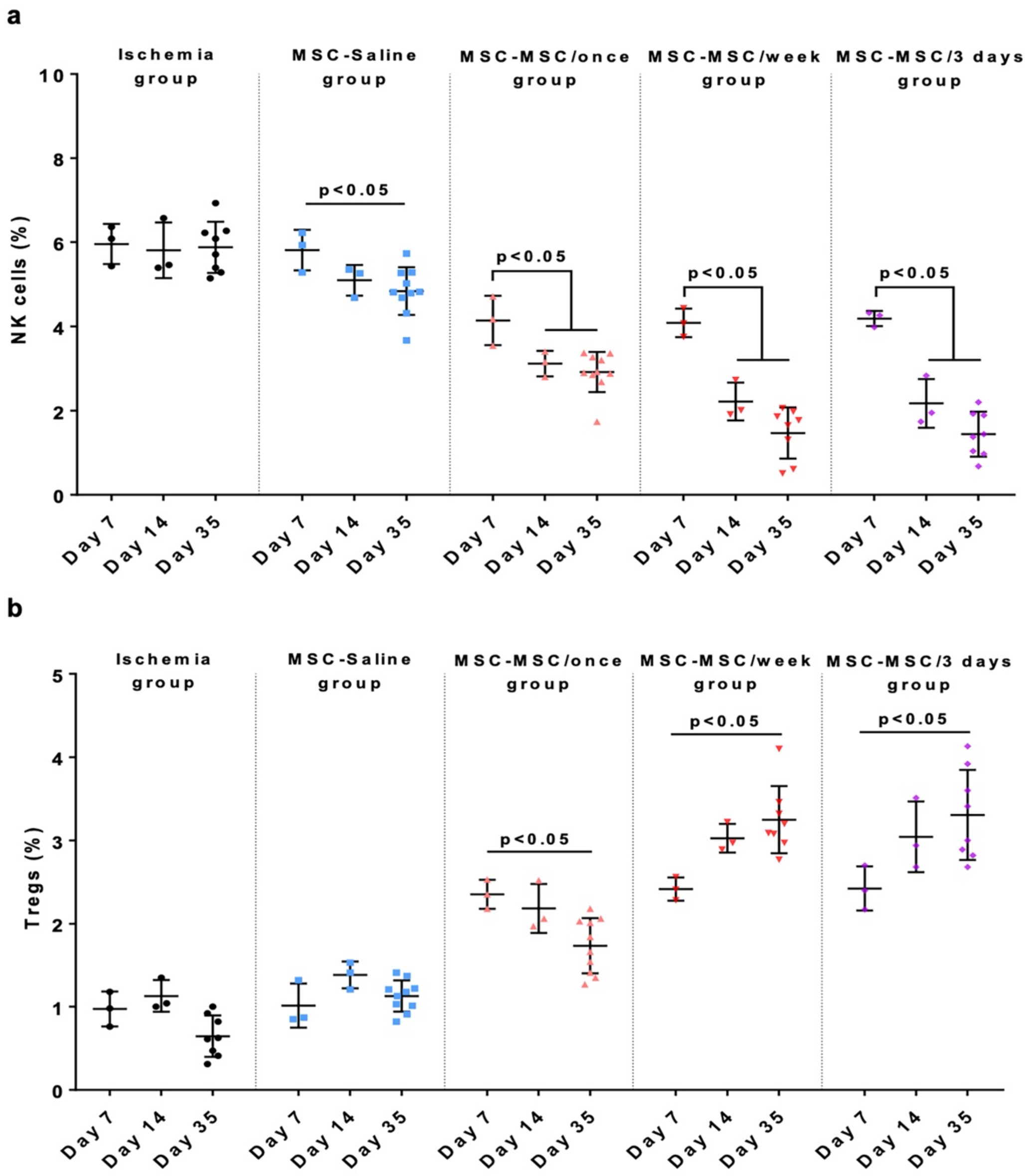

The changes of splenic Tregs and NK cells within each group were showed in this figure. Splenic NK cells progressively decreased following intramuscular hiPSC-MSCs transplantation or intravenous hiPSC-MSCs infusion in MSC-Saline, MSC-MSC/once, MSC-MSC/week and MSC-MSC/3 days groups, whereas no significant difference between different time points in ischemia group (**a**). Splenic Tregs progressively decreased after reached the peak level at day 7 in MSC-MSC/once group, whereas these immunomodulatory cells continued to increase in MSC-MSC/week and MSC-MSC/3 days groups. No significant difference between different time points in ischemia and MSC-Saline groups (**b**).

Supplementary Figure 5. The cell survival and engraftment have no difference between subcutaneous administration of cyclosporine A and single or repeated intravenous hiPSC-MSC infusion.

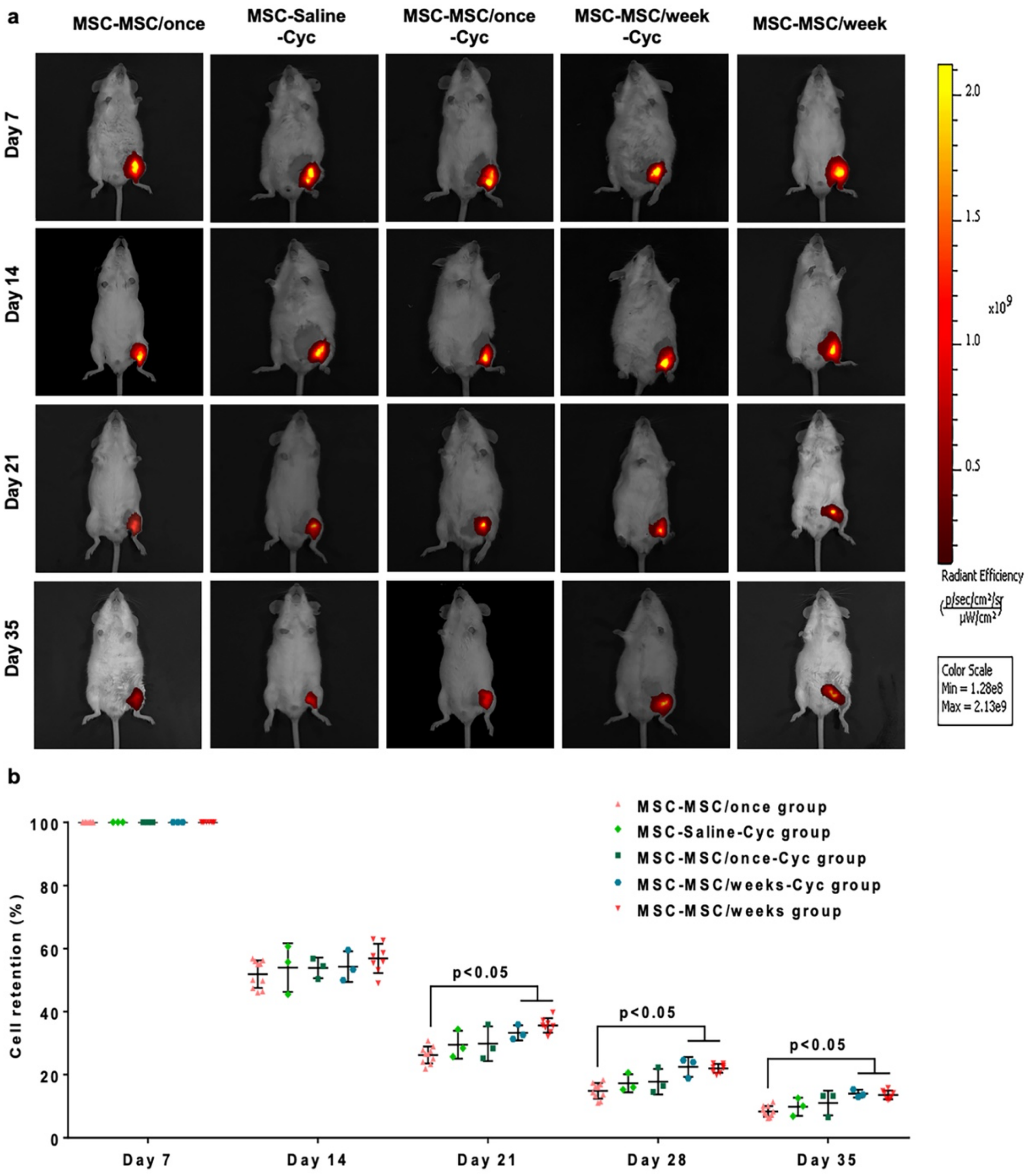

To evaluate cell engraftment in the groups that received daily subcutaneous administration of cyclosporine A treatment, a series of fluorescent images of ischemic hind limb was obtained immediately and every week following intramuscular transplantation of hiPSC-MSCs **(a)**. There was no significant difference among the MSC-MSC/once, MSC-Saline-Cyc and MSC-MSC/once-Cyc groups. Repeated intravenous infusion of hiPSC-MSCs with or without subcutaneous administration of cyclosporine A significantly increased cell engraftment compared with the MSC-MSC/once group (**b**).

Supplementary Figure 6. Subcutaneous administration of cyclosporine A did not improve the blood perfusion without intravenous hiPSC-MSC infusion.

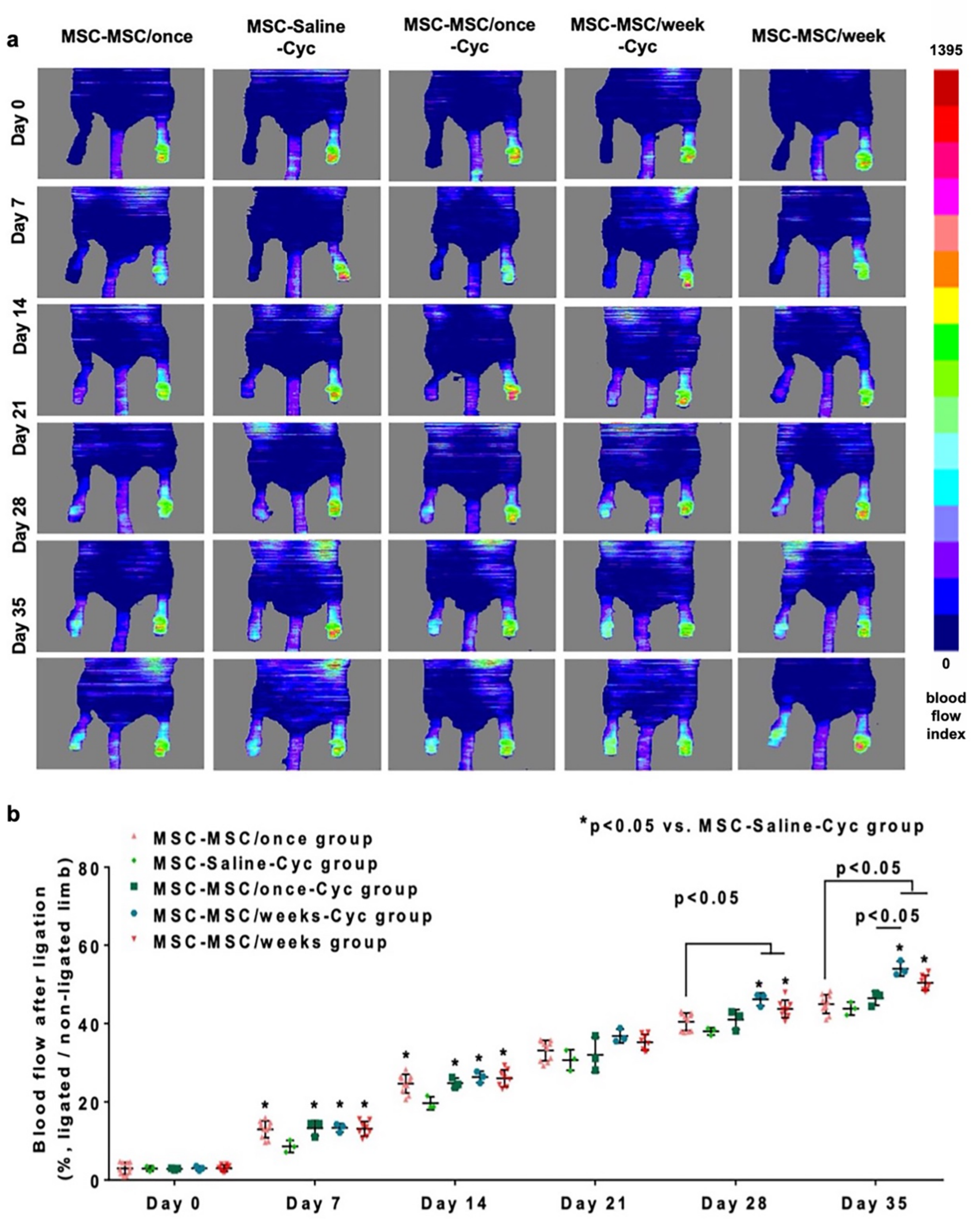

To evaluate blood perfusion in the groups that received subcutaneous administration of cyclosporine A, Laser Doppler imaging analysis was performed immediately and every week following femoral artery ligation **(a)**. A single intravenous administration of hiPSC-MSCs in the MSC-MSC/once, MSC-MSC/week, MSC-MSC/once-Cyc or MSC-MSC/week-Cyc groups significantly increased blood perfusion during first two weeks compared with the MSC-Saline-Cyc group. Nonetheless, by the end of experiment, no

significant difference was observed among the MSC-MSC/once, MSC-Saline-Cyc and MSC-MSC/once-Cyc groups. Repeated intravenous infusion of hiPSC-MSCs with or without subcutaneous administration of cyclosporine A significantly increased therapeutic efficacy relative to the MSC-MSC/once group (**b**).

Supplementary Figure 7. The optimal time and dosage for systemic intravenous hiPSC-MSC infusion.

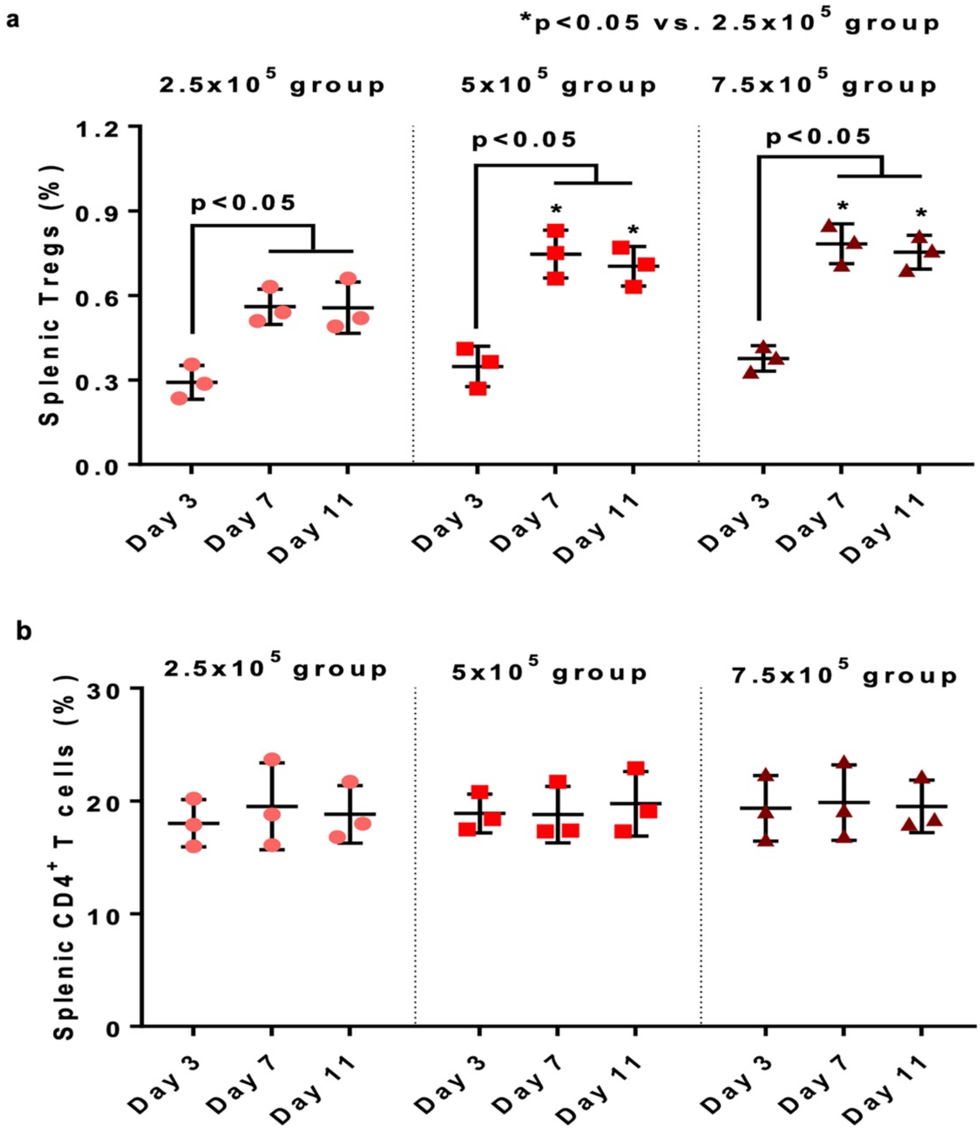

To define the optimal time and dosage for systemic intravenous hiPSC-MSC infusion, we compared the changes of splenic Tregs after a single intravenous administration of $2.5x10^5$, $5x10^5$ or $7.5x10^5$ hiPSC-MSCs in the $2.5x10^5$, $5x10^5$ or $7.5x10^5$ groups respectively. The results showed that after a single intravenous hiPSC-MSC infusion in the $2.5x10^5$, $5x10^5$ or $7.5x10^5$ groups, splenic Tregs progressively increased to their peak level at day 7. When comparison among $2.5x10^5$, $5x10^5$ and $7.5x10^5$ groups was performed, higher splenic Tregs level was observed in the $5x10^5$ or $7.5x10^5$ groups compared with the $2.5x10^5$ group. There was no significant difference between the $5x10^5$ and $7.5x10^5$ groups (**a**). There was no significant difference in splenic $CD4^+$ T cells within or between all three groups (**b**).

Supplementary Figure 8. FACS gating strategy.

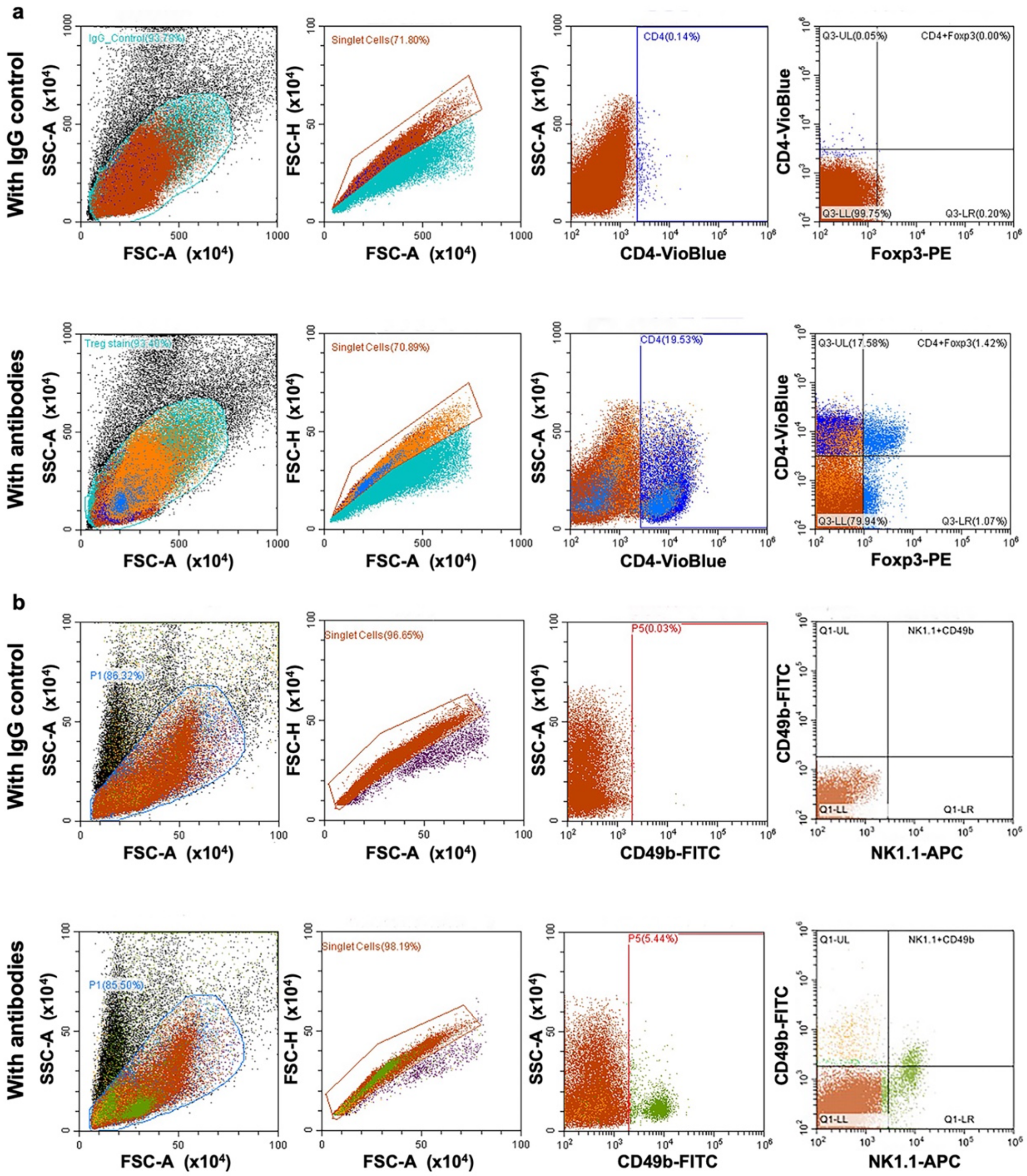

Splenocytes staining with IgG were used as control for gating strategy. The number of Tregs was expressed as Foxp3^{+} cells in a proportion of pre-gated CD4^{+} cells (**a**). The number of NK cells was expressed as CD49b^{+} cells in a proportion of pre-gated NK1.1^{+} cells (**b**).
