# Supplementary material for: Repeated intravenous administration of hiPSC-MSCs enhance the efficacy of cell-based therapy in tissue regeneration
Source: Commun Biol. 2022 Aug 25;5:867. doi: 10.1038/s42003-022-03833-8 (PMC9411616; doi:10.1038/s42003-022-03833-8)
Supplement: Supplementary file 3 — Description of Additional Supplementary Files [file 42003_2022_3833_MOESM3_ESM.pdf]

Description of Additional Supplementary Files

File name: Supplementary Data 1
Description: The estimated survival rates in MSC-Saline, MSC-MSC/once, MSC-MSC/week and MSC-MSC/3 days groups.
