# Supplementary material for: Repeated intravenous administration of hiPSC-MSCs enhance the efficacy of cell-based therapy in tissue regeneration
Source: Commun Biol. 2022 Aug 25;5:867. doi: 10.1038/s42003-022-03833-8 (PMC9411616; doi:10.1038/s42003-022-03833-8)
Supplement: Supplementary file 5 — Reporting Summary [file 42003_2022_3833_MOESM5_ESM.pdf]

nature portfolio

Corresponding author(s): Song-Yan Liao

Last updated by author(s): 07/07/2020

Reporting Summary

Nature Portfolio wishes to improve the reproducibility of the work that we publish. This form provides structure for consistency and transparency in reporting. For further information on Nature Portfolio policies, see our Editorial Policies and the Editorial Policy Checklist.

Statistics

For all statistical analyses, confirm that the following items are present in the figure legend, table legend, main text, or Methods section.

n/a	Confirmed	
☐	☒	The exact sample size (*n*) for each experimental group/condition, given as a discrete number and unit of measurement
☐	☒	A statement on whether measurements were taken from distinct samples or whether the same sample was measured repeatedly
☐	☒	The statistical test(s) used AND whether they are one- or two-sided<br>*Only common tests should be described solely by name; describe more complex techniques in the Methods section.*
☒	☐	A description of all covariates tested
☐	☒	A description of any assumptions or corrections, such as tests of normality and adjustment for multiple comparisons
☐	☒	A full description of the statistical parameters including central tendency (e.g. means) or other basic estimates (e.g. regression coefficient) AND variation (e.g. standard deviation) or associated estimates of uncertainty (e.g. confidence intervals)
☐	☒	For null hypothesis testing, the test statistic (e.g. *F*, *t*, *r*) with confidence intervals, effect sizes, degrees of freedom and *P* value noted<br>*Give P values as exact values whenever suitable.*
☒	☐	For Bayesian analysis, information on the choice of priors and Markov chain Monte Carlo settings
☒	☐	For hierarchical and complex designs, identification of the appropriate level for tests and full reporting of outcomes
☒	☐	Estimates of effect sizes (e.g. Cohen's *d*, Pearson's *r*), indicating how they were calculated

Our web collection on statistics for biologists contains articles on many of the points above.

Software and code

Policy information about availability of computer code

Data collection	n/a
Data analysis	n/a

For manuscripts utilizing custom algorithms or software that are central to the research but not yet described in published literature, software must be made available to editors and reviewers. We strongly encourage code deposition in a community repository (e.g. GitHub). See the Nature Portfolio guidelines for submitting code & software for further information.

Data

Policy information about availability of data

All manuscripts must include a data availability statement. This statement should provide the following information, where applicable:

- Accession codes, unique identifiers, or web links for publicly available datasets
- A description of any restrictions on data availability
- For clinical datasets or third party data, please ensure that the statement adheres to our policy

All the data are available from the corresponding authors upon reasonable request.

Field-specific reporting

Please select the one below that is the best fit for your research. If you are not sure, read the appropriate sections before making your selection.

☒ Life sciences ☐ Behavioural & social sciences ☐ Ecological, evolutionary & environmental sciences

For a reference copy of the document with all sections, see nature.com/documents/nr-reporting-summary-flat.pdf

Life sciences study design

All studies must disclose on these points even when the disclosure is negative.

Sample size	We used commercial available software G Power for sample size calculation.
Data exclusions	Animals with foot necrosis in the ischemia group were excluded from the analysis (n=2) due to administration of a large bolus of pain relief and antibiotic agents that may affect the results of flow analysis.
Replication	All the measurement were replicated three time and the mean of these data was collected.
Randomization	After animal model was established, all animals were randomized into different group.
Blinding	All the measurement was collected by two different observers who are blinded to the experiment group.

Reporting for specific materials, systems and methods

We require information from authors about some types of materials, experimental systems and methods used in many studies. Here, indicate whether each material, system or method listed is relevant to your study. If you are not sure if a list item applies to your research, read the appropriate section before selecting a response.

Materials & experimental systems

n/a	Involved in the study	
☐	☒	Antibodies
☒	☐	Eukaryotic cell lines
☒	☐	Palaeontology and archaeology
☐	☒	Animals and other organisms
☒	☐	Human research participants
☒	☐	Clinical data
☒	☐	Dual use research of concern

Methods

n/a	Involved in the study	
☒	☐	ChIP-seq
☐	☒	Flow cytometry
☒	☐	MRI-based neuroimaging

Antibodies

Antibodies used	anti-human mitochondria antibody (1:100, MAB1273, Merck Millipore, CA, USA); anti-human GAPDH antibody (1:100, ab128915, Abcam, MA, USA); anti-mouse α-SMA (1:200, M0851, Sigma-Aldrich, MO, USA); anti-mouse vWF (1:200, ab7356, Merck Millipore, CA, USA); anti-mouse CD68 (1:200, ab31630, Abcam, MA, USA); anti-Arginase 1 (1:100, ab60176, Abcam, MA, USA).
Validation	All the validation statement can be found on manufacturer's website.

Animals and other organisms

Policy information about studies involving animals; ARRIVE guidelines recommended for reporting animal research

Laboratory animals	ICR mouse, male, aged 12-16 weeks.
Wild animals	This study did not involve wild animals.
Field-collected samples	All animals were euthanized by intraperitoneal injection of 100mg/kg pentobarbital. Fresh splenocytes were isolated for folw cytometry analysis. All the ischemic hindlimbs were collected for histological study.
Ethics oversight	All procedures that involved animals were approved by the Committee on the Use of Live Animals in Teaching and Research (CULATR) at the University of Hong Kong. (CULATR number 4777-18)

Note that full information on the approval of the study protocol must also be provided in the manuscript.

Flow Cytometry

Plots

Confirm that:

☒ The axis labels state the marker and fluorochrome used (e.g. CD4-FITC).

☒ The axis scales are clearly visible. Include numbers along axes only for bottom left plot of group (a 'group' is an analysis of identical markers).

☒ All plots are contour plots with outliers or pseudocolor plots.

☒ A numerical value for number of cells or percentage (with statistics) is provided.

Methodology

Sample preparation	After animals were sacrificed, the fresh spleen was harvested and washed with phosphate buffered saline (PBS). A single cell suspension of splenic lymphocytes was prepared by meshing through 100 µm nylon cell strainers (Falcon, NY, USA) and red blood cells removed by ACK lysing solution (A1049201, Invitrogen, CA, USA).
Instrument	Cytomic FC500 flow cytometer (Beckman Coulter, cytoflex-STM 682 , FL, USA)
Software	FlowJo software (V10.7.1, Becton, USA)
Cell population abundance	One million splenocytes were then blocked with purified anti-mouse CD16/32 antibody (1:100, 101302, BioLegend, CA, USA) and 7AAD (1:50, 559925, BD Bioscience, NJ, USA) in flow buffer containing PBS, 1% BSA, 2 mM EDTA, 0.01% NaN3 for 10 min at 4 ℃.
Gating strategy	IgG antibodies without labeling were used for gating strategy.

☒ Tick this box to confirm that a figure exemplifying the gating strategy is provided in the Supplementary Information.
